# Reasons for never and intermittent completion of colorectal cancer screening after receiving multiple rounds of mailed fecal tests

**DOI:** 10.1186/s12889-017-4458-6

**Published:** 2017-05-30

**Authors:** Beverly B. Green, June BlueSpruce, Leah Tuzzio, Sally W. Vernon, L. Aubree Shay, Sheryl L. Catz

**Affiliations:** 1 Kaiser Permanente Washington Health Research Institute, Metropolitan Park East, 1730 Minor Avenue Suite 1600, Seattle, WA 98101 USA; 2 University of Texas Health Science Center Houston, School of Public Health, 1200 Pressler Street, Houston, TX 77030 USA; 3 Betty Irene Moore School of Nursing, University of California Davis, 1 Shields Avenue, Davis, CA 95616 USA

**Keywords:** Colorectal cancer screening, Fecal testing, Adherence, Qualitative research, Barriers and facilitators

## Abstract

**Background:**

Long-term adherence to colorectal cancer (CRC) screening is particularly important for fecal testing. Some U.S. studies report that only 25% of individuals repeat fecal testing annually. The purpose of this qualitative study was to identify barriers and facilitators reported by patients with suboptimal screening adherence to refine interventions for starting ongoing adherence to CRC screening. We also explored whether participants, particularly never screeners, would be willing to do a CRC screening blood test.

**Methods:**

Forty-one patients who previously enrolled in the Systems of Support to Increase CRC Screening (SOS) trial were interviewed 4–5 years later. Participants were purposively selected to include men and women with diverse race/ethnicities who had either been inconsistent screeners or had never screened during the first three years of SOS despite receiving at least two rounds of mailed fecal tests. Two interviewers conducted 30-min telephone interviews using a semi-structured interview guide. An iterative thematic analysis approach was used.

**Results:**

Themes related to screening barriers were more pervasive among never screeners including: (1) Avoidance (inattention, procrastination) (2) Concerns about handling stool; (3) Health concerns; (4) Fear of a cancer diagnosis or positive test results. Themes related to screening facilitators were more often mentioned by participants who screened at least once including: (1) Use of a simpler 1-sample fecal test; (2) Convenience of mailings and doing the test at home; (3) Salience of prevention, especially as one got older; and (4) Influence of recommendations from providers, family and friends. Participants had diverse preferences for the number (3 on average) and types (phone, mail, text) of screening reminders. Some participants did not prefer e-mail links to the patient shared electronic health record because of difficulties remembering their password. It was acceptable for a nurse or medical assistant not from their clinic to call them as long as that person was knowledgeable about their records and could communicate with their physician. Participants, especially never screeners, were generally very enthusiastic about the potential option of a CRC screening blood test.

**Conclusion:**

Future CRC screening programs should be designed to minimize these barriers and maximize facilitators to improve long-term screening adherence.

**Trial registration:**

Primary Funding Agency: The National Cancer Institute of the National Institutes of Health (R01CA121125). Registered at clinicaltrials.gov NCT00697047.

## Background

The lifetime risk of colorectal cancer (CRC) is over 5% [1] in the United States. Despite recent declines in incidence and mortality, CRC remains the second highest cause of cancer death [1]. Better treatments have improved survival rates, but morbidity and mortality could be reduced more rapidly and cost-effectively by achieving higher uptake and long-term adherence to CRC screening [2]. Despite this, screening rates remain suboptimal, with only 62% of age-eligible adults current for CRC screening in the U.S. [3].

The US Preventive Services Task Force recommends CRC screening with colonoscopy every 10 years, fecal testing (using a high-sensitivity guaiac or fecal immunochemical test [FIT]) annually, flexible sigmoidoscopy every 10 years combined with annual fecal testing, virtual colonoscopy every 5 years, or a combination of DNA and fecal testing every 1 to 3 years [4]. Almost all trials testing interventions to increase CRC screening rates have tested the effect of an intervention on only a single round of screening. Only a few trials have evaluated repeated interventions and factors related to ongoing adherence, which is particularly important for individuals choosing fecal testing, with completion recommended annually [5, 6]. Large population-based screening programs provide additional information about long-term adherence to multiple rounds of mailed FOBT. In Scotland, among 251,578 eligible adults, adherence of 55%, 45%, and 48% in years 1, 2, and 3, respectively, were reported [7]. In the Netherlands, among 23,339 participants, adherence to 3 rounds of biennial FIT ranged from 60% to 63%, with 72% participating at least once and 48% participating in all rounds [8].

Systems of Support to Increase Colorectal Cancer Screening (SOS) is an ongoing four-arm randomized trial that compares usual care (arm-1) to stepped intensity interventions that include more resources for each step: mailings alone (arm-2, a pamphlet about CRC screening choices, a number to call if colonoscopy or flexible sigmoidoscopy was preferred, and mailed fecal kits for those not calling); mailings plus telephone-based brief assistance (arm-3); or mailings, brief assistance, and more intense telephone-based nurse navigation [9]. Adherence to CRC screening over two years of the study was more than double that of usual care (51% to 65% depending on intervention intensity vs. 26% in usual care; *P* < .001) [10]. Despite the fact that the interventions were effective, over 20% of active intervention individuals did not complete a CRC screening test even though they were offered the option to complete colonoscopy or flexible sigmoidoscopy and mailed at least two fecal test kits.

The mailed interventions were effective for all participant subgroups by age, sex, race, ethnicity, education level, prior-screening, and self-rated health [10]. However, response to the stepped interventions varied, by subgroup with African Americans less responsive to the brief telephone support and smokers being somewhat less likely to repeat fecal testing in year 2 of the study. Additionally over 20% of individuals offered stepped interventions did no CRC screening test despite being offered at least 2 rounds of mailings and for arms 3 and 4 stepped intensity phone support.

SOS interventions were based on the Preventive Health Model (PHM), which in part is based on the Health Belief Model [11, 12]. According to the PHM, CRC screening completion is mediated by an individual’s beliefs about the pros and cons of screening and specific tests, perceived risk for CRC, social influence (such as a doctor’s or family member’s recommendation), self-efficacy for completing testing, and prior testing experience. However, in analysis of surveys conducted pre randomization and post year 2 interventions, we found that the only PHM constructs that predicted screening were prior completion of CRC tests and self-efficacy [13]. The strongest predictor of CRC screening initiation and repeat testing was receiving active interventions. Because there were still many individuals who remained unscreened or screened intermittently and because the PHM did not fully predict screening uptake and adherence, we wanted to explore emergent themes and participants’ experiences for the purpose of informing intervention refinements.

In our review of qualitative studies of barriers and facilitators to CRC screening, the published literature focused primarily on initial uptake of screening, with few studies looking at factors related to repeat screening. In a study of Kaiser Permanente Northern California patients offered 3 rounds of mailed FIT, Gordon and Green compared patients who completed all rounds (continuers), those who completed a kit only in year 3 (converters), and those that completed no kits (non-screeners) [14]. Converters more frequently cited guilt and pleasing their doctor as reasons for screening, while non-screeners more frequently reported discomfort, disgust, or embarrassment handling stool and the belief that screening was not necessary. Duncan et al. surveyed Australians offered 3 rounds of FIT screening and reported low self-efficacy and less satisfaction with screening as predictors of non-screening, with male sex and younger age as predictors of delayed screening [15]. Neither of these studies performed in-depth interviews with patients to explore factors related to late initiation or repeated refusal of CRC screening after being offered multiple rounds of fecal tests and mailed and phone information on its importance. We undertook this qualitative study to further refine the SOS study and to understand key factors that might get non-screeners to start screening, and irregular screeners to adhere to recommended screening intervals. Recently the Food and Drug Administration (FDA) approved a CRC screening blood test, so we also explored participants’ perception of this test for initiation and ongoing screening adherence [16].

## Methods

### Setting and participants

This qualitative study is part of Systems of Support to Increase Colorectal Cancer Screening Trial (SOS) supported by a grant from the National Cancer Institute (R01CA121125) and conducted at Group Health Cooperative, a nonprofit, integrated health care system serving almost 600,000 members in Washington State. At the time of enrollment into the SOS study (August 2008 to November 2009), participants were 50–73 years of age and not current for CRC screening by national guidelines; almost half (46%) had never done any type of CRC screening test.

Participants for this qualitative study were drawn from the subset of SOS participants who had been randomized to receive active interventions, which included a mailed pamphlet about CRC choices (colonoscopy, flexible sigmoidoscopy, and fecal testing pros and cons, recommended intervals), a phone number to call if they preferred colonoscopy or flexible sigmoidoscopy, and FIT kits mailed to participants. Few people called the provided phone number, and almost all participants in the active intervention groups received mailed fecal kits. Some active intervention participants who did not screen after they received the mailed interventions received further support to complete CRC screening (brief phone assistance from a medical assistant, or this plus nurse navigation). A full description of the study design and results of the initial 2 year and subsequent 3 year interventions have been published [9, 10, 17].

Among active intervention participants, we identified participants who had never been screened or had completed only one fecal screening test over the first 3 years of the study and no colonoscopy or flexible sigmoidoscopy despite being mailed at least 3 fecal tests. We did purposive sampling, oversampling by male sex, race and smoking status to assure representation by subgroups somewhat less likely to complete CRC screening in our trial despite stepped intensity phone support (e.g., African Americans, smokers). All participants had been offered at least 2 years of mailed fecal tests and a pamphlet describing alternative tests (colonoscopy and flexible sigmoidoscopy) and a number to call if they preferred these tests; some participants had received more intensive phone support to complete either fecal testing or, if they preferred, colonoscopy or sigmoidoscopy.

Potential participants were contacted by phone, and those who provided verbal consent were interviewed. Two investigators (JB, LT) used a semi-structured interview guide with 16 questions and probes to encourage in-depth exploration of each issue. In the initial SOS study, we found that the PHM was only modestly predictive of initial and repeat screening [11]. Due to this finding, we decided to explore potential alternative mediators to complete CRC screening. The interview guide included questions about new topics such as a hypothetical blood test, knowledge about screening intervals for the different cancer screening tests, and factors that prevent or delay someone from completing screening, as well as facilitators and barriers (test pros and cons, social influence, risk, avoidance and procrastination, optimism) and the influence of these factors on repeat screening (Appendix).

Two investigators (JB, LT) blinded to the screening status (i.e., whether the patient was a never or one-time screener during the first three years of the SOS study) conducted telephone interviews with 41 Group Health members between December 2013 and February 2014. Interviews were 0.5–1 h long and were audio-recorded with respondents’ permission. We paid participants $30 for completing the interview. A professional transcriptionist transcribed the interviews verbatim. The investigators reviewed their transcripts for accuracy and filled in inaudible passages. The Group Health Institutional Review Board (IRB) approved all aspects of this study.

### Qualitative analysis

Three investigators (SC, JB, LT) analyzed transcripts using a descriptive thematic approach [18]. First, one analyst (JB) proposed categories and subcategories based on the interview questions (Appendix). She used a subset of 11 interview transcripts to validate these categories and develop a list of specific codes. All three investigators then discussed and revised the list and developed code definitions. Two investigators (JB, LT) coded five transcripts and compared coding. They continued to code a total of 10 transcripts, compare, resolve discrepancies, and clarify codes and definitions until they reached 80% agreement. The coders completed coding all transcripts without further comparison. Our coding was descriptive and iterative, identifying many themes [18]. Atlas.ti® software was used for data management and coding (Version 7.0, Berlin, Germany).

The three investigators summarized findings according to salient themes across all interviews with supporting quotes. For subgroup analysis, after all coding had been completed and categorized, we assessed the relevant themes by the participant’s original sampling group (never or infrequent screeners). We also assessed the theme by whether the original non-screeners remained a non-screener (*never screened*), became a screener after year 3 but prior to the interviews (*converters*), were one-time screeners who did not repeat screening after the initial FIT test (*stopped*) or screened again after year 3 (*repeaters*). We identified the most commonly mentioned themes and highlighted some of the cross-cutting themes by sub-group (e.g., African-American, smokers).

## Results

Of the 113 SOS participants we attempted to contact, 41 completed interviews (36%). We were unable to contact 49, 10 opted out of participation, 4 were ineligible because they had left Group Health, and 9 were not interviewed because of scheduling difficulties. Of the 41 interviewed participants, 18 were male (44%), 12 were African American (30%), and 10 were current smokers (25%) compared to our sampling goals of 50% male, 30% African-American, and 30% smokers (Table 1).

**Table 1:** Qualitative Study Sample^a^ – Based on Screening Participation in the First Three Years of the Systems of Support to Increase Colorectal Cancer Screening Study (SOS)

	Initial non-Screeners^b^	Initial one-time screeners^c^
	*N* = 18 (44%)	*N* = 23 (56%)
Sex		
Male	8 (44)	10 (43)
Female	10 (56)	13 (57)
Race		
White or any other race besides African-American	14 (78)	15 (65)
African-American	4 (22)	8 (35)
Smoking status		
Never/prior smoker/unknown	12 (67)	19 (83)
Current smoker	6 (33)	4 (17)
Completed CRC screening after year 3 of the study		
No	13 (72)^d^	5 (21)^e^
Yes	5 (28)^f^	18 (79)^g^

^a^All participants were enrolled in the SOS study and received at least two rounds of mailed FIT tests

^b^Non screeners were defined as those who completed no CRC test in the first three years of the study

^c^Intermittent screeners were defined as those who completed 1 fecal test in the first three years of the study

^d^Initial non-screeners who did no CRC tests in the first three years and no tests after are called *Never* screeners

^e^Initial one-time screeners who did one CRC test in the first three years but no subsequent tests are called *Stopped* screeners

^f^Initial non-screeners who did no CRC tests in the first three years and 1 CRC test after year 3 are called *Converting* screeners

^g^Initial one-time screeners who did one CRC test in the first three years and repeated tests after are called *Repeating* screeners

Among the 41 participants that completed interviews, 23 completed one fecal test and 18 did no fecal tests in the first 3 years of the SOS study, despite being offered at least 2 rounds of mailings with fecal kits (Table 1). The majority of the one-time screeners (18/23 (78%)) repeated screening in year 4 or 5, while 5/23 (22%) stopped and did not screen again. Most of the initial non-screeners (13/18 (72%)) remained never screened with only 5/18 (28%) converted, or screened for the first time after year 3.

### Barriers to CRC screening

By far, the most frequently mentioned category of barriers to FIT and other CRC screening tests related to not paying attention, remembering, taking time for the test, or procrastinating about doing it (collectively referred to as *Avoidance* based on work by McQueen et al., [19]) (Table 2). *“It’s like I have 17 things to do on my to-do list in a day, and I never get more than eight of them done and it’s a matter of propelling it up into the top eight,”* or *“Out of sight, out of mind.”* Procrastination was mentioned mostly by those who had screened, with one converter commenting that once they had actually completed a FIT, they wondered why they had put off testing for so long.

**Table 2:** Barriers to colorectal cancer screening^a^

	*Never* screeners (*N* = 13)	*Stopped* -screened once before Year 3 (*N* = 5)	*Converted* - screened once after year 3 (*N* = 5)	*Repeated* - screened once before year 3 and at least once after (*N* = 18)
Avoidance	*“That know we should, but don’t do it anyway? It’s like I have 17 things to do on my to-do list in a day, and I never get more than eight of them done and it’s a matter of propelling it up into the top eight.”*	*“I probably wait a little bit. I probably get them and then don’t think about them right away. Usually my wife will find them and say, are you going to do this?”* *“You don’t want to think about it.”*	*“I think if I recall, when I did it, it kind of wasn’t such a big deal. Almost like shaking my finger at myself. Why did you put it off so long? I do know that when I decided to do it and I set aside the time to do it, there was not a sense of urgency that I had.”*	*“I’d not been actually taking care of myself, that guy thing of ignoring the doctors and all… Basically just ignoring the fact that I was getting older.”*
Test Specific Barriers	*“I don’t want to test my poo at all, I don’t want to go anywhere near it. I want it in the toilet getting flushed. I just don’t want to deal with that part of it at all, that’s what I found disgusting about the whole thing was that you had to get* samples of it to send in.” “*Just the idea of putting that in the mail somehow bothers me. Back when they were having problems with people supposedly sending anthrax in the mail, I was kind of almost horrified that you can send that in the mail and I was thinking it doesn’t seem sanitary…”* *“Well, I didn’t go in and have a colonoscopy or anything like that. But I thought about it. But everybody I talked to, they said it hurts real bad.”*	*“I could probably produce a stool sample and take it in to them and let them deal with whatever comes after [that], but me having to poke the little sticky thing into it and put it in the bottle? That was very difficult for me.”* *“I have a really strong gag reflex, I came really close to throwing up, but I did it anyway.”* *“So I was thinking how in the world can someone go through these steps, go through this test [colonoscopy], and have it done comfortably? It would really help to make it a little less, I don’t know, arduous preparation?”*	*“Most of the time as an adult, from the time you’re a child, you were told not to play with your stool. Now here you are, you’re an adult and all these years have gone by where you’ve never played with your stool, and somebody’s asking you to play with your stool.”* *“I know I was supposed to have a complete colonoscopy, but with my work schedule I couldn’t get that much time off to where - the day before you drink all the goop, you got to have that one and then the day of.”*	*“…it’s hard to put into words, but my best description of it is when you actually wipe yourself and you take a sample from it… and go through the procedure, following the basic steps as far as putting [it] in the little vial… It’s a little awkward.”* *“They have you lay out the paper in the bowl and then just take the sample right off the paper. The only concern is it going to fall off the paper or whatever. But I’ve never had a problem with it so it’s not a concern anymore.”* *“Having to flush yourself out for a few days, you do that and the day before, on the pot, on the toilet. Then you go in and the only thing you can think about the whole time is you’re starving. That’s about the size of it [colonoscopy].”*
Fear	*“Initially, I didn’t really understand it as a screening. For whatever reason ... I was thinking... if it comes up positive they’re going to whisk me into surgery or something.”* *“I think people who are African American you have to let them know it’s not nothing scary, because we’re kind of leery and afraid, so information would be good.”*		*“I’m a positive person primarily and I probably would not want to introduce something into my world that’s negative. If I only have a month to live, I’d rather not know about it for about three weeks.”*	*“It’s not I would fear what the results might be, that’s one thing about it. I fear they might discover something, and then on the flipside of the coin I fear that if I don’t have it [colonoscopy] and there is something, that I waited too long.”*
Health Concerns	*“I just haven’t been able to really – with my irritable bowel syndrome, it’s really hard for me to even – some days I don’t produce a stool...”* *“Yeah, I deal with diabetes, COPD… I got some other health issues. They kind of coincide and swirl around each other, and that pulls me down… I know it’s my weight. If I could get my weight off, I’d feel a ton better. I gained weight too because I’ve been sick. The doctor says it’s like sitting in the middle of a spinning circle…Just one big thing after another, going around and around my body.”*	*“Well, the reason that I haven’t in the past is if I have hemorrhoids really bad, they’re bleeding. ….Other than that, it would be no problem.”*		*“I’ve reached the point now – this is a foot injury which causes some side effects of using the toilet, and now I’m reaching the point where I can actually do the sample without risking injury.”*

^a^Within the context of receiving a pamphlet about colorectal cancer screening choices and at least two mailed fecal tests

Barriers were reported more frequently by never screeners, and they used language that was more emphatic. This included *Cons of a Specific Test*, particularly aversion to handling stool, with statements like *“I don’t want to test my poo at all, I don’t want to go anywhere near it. I want it in the toilet getting flushed.”* In contrast, when dislike of stool was mentioned by a repeated screener, it tended to be more matter-of-fact: “*You take a sample from it… and go through the procedure, following the basic steps as far as putting [it] in the little vial… It’s a little awkward.”* Barriers to completing fecal testing also included difficulties with avoiding meat for the stool guaiac test. Barriers related to completion of colonoscopy (prep, taking off work, concerns about anesthesia) were mentioned frequently. Study participants were all offered an option to do colonoscopy instead of fecal tests, but few requested this, and all got mailed kits. However a few participants mentioned that colonoscopy was the most accurate test. One stated that the difficulties of getting a colonoscopy done had paradoxically led them to avoid any testing: *“Probably because I've been thinking on terms that I've got to have the colonoscopy done anyways, so I'm looking at it that way instead of saying oh, do the stool test, when it's the colonoscopy I have to get through.”*

*Fear* of finding out about a cancer diagnosis and fear in general was also mentioned, with a never screener stating *“I was thinking... if it comes up positive they're going to whisk me into surgery or something.”* Fear and avoidance appeared to interact with each other; one stopped screener stated that the risk of CRC did not motivate him (*“I don’t want to think about it”).*

*Health-Related Issues* were mentioned mostly by never screeners as reasons not to get screened such as concerns about medications, like warfarin, diarrhea, or having other chronic conditions including depression. A never screener listed multiple health issues that made it more difficult to get screened: “*I have diabetes, I have heart issues. I can't tell you how many different medications I'm on... I have to prioritize my needs.”* Of the nine participants who mentioned other physical conditions or symptoms as a barrier to FIT testing, five of them remained never screeners, suggesting this was an important impediment to screening.

### Facilitators for CRC screening

The most commonly mentioned facilitator to completion of screening was the convenience of testing, being able to do it in the privacy of one’s home. This facilitator was mentioned in reference to the one-time fecal test (FIT) compared to the three-sample guaiac fecal test (*Pros of a Specific Test*) (Table 3). While convenience was mentioned mainly by those that had completed at least one fecal test, it was also mentioned occasionally by never screeners, but more hypothetically (*“this one sounds so much better”*). Getting the fecal test in the mail (part of the SOS intervention program) also helped facilitate screening. *“You get it in the mail and then you… follow the instructions, take a sample and mail it in and you get your results online. It’s real easy…it takes a minute, literally one minute to do, and then you’re done for a year, you don’t have to go in to the doctor or anything”* (repeater).

**Table 3:** Facilitators for Colorectal Cancer Screening^a^

	*Never* screeners (*N* = 13)	*Stopped* - screened once before year 3 (*N* = 5)	*Converted* - screened once after year 3 (*N* = 5)	*Repeated* - screened once before year 3 and at least once After (*N* = 18)
Test Specific Facilitators	*“[FOBT] was a little hard. Because you had to watch your diet and you had to do everything. This one sounds – I haven’t done it yet, but [*FIT] *sounds so much better.”* *“I don’t know how long they’ve been doing this, but if they had some kind of brief update as far as how effective the test was. So, for example, if they did screening and X percentage of the screened samples were positive and then out of those that were positive, they do follow up and X percentage of those were found to be accurate or something like that. Demonstrate the effectiveness of the screening.”*	*“They gave me a new type of product for [the] test, which you don’t have to have a specific diet to do anymore, so ...not as complicated as having to wait three days without eating certain products…It was easy, it really wasn’t handling anything, you just collected it in a swab and stuck it in a tube rather than having to spread it on a card...”* *“Well, first of all, it’s kind of a personal body function sort of thing so the sooner I could do the test and get over with it and hear the results, I would be happier….So if I could do one thing and be tested, that would be great. That would be my opinion. The quicker and the more complete the test could be, that would be great.”*	*“That [one-sample test] makes it much more appealing.”* *“That one was easy because you didn’t have to watch what you ate or nothing like that, you just did the sample and then sent it in which was really easy compared to when my husband did one years ago, where he couldn’t eat certain kind of food for so many days and then do it.”*	*“Well, what really kind of helped turn things around, I was really impressed – but an easier test…the package came with a system that was much more doable and much clearer in the instructions. None of this, three blank pieces of cardboard that you have to swipe and that crap…the testing procedure itself was greatly improved.”* *“And then the last time when I went in for an exam, they said here’s one, take it, it’s new, it’s easier to do, the latest test for the bowel. It was a lot easier. It’ll be quicker and you can either bring it in or mail it in…It didn’t have as many steps, and the restrictions from what you could eat and not eat - because I would just forget, and think I’m going to do it today and then oh, I can’t because I ate red meat. So the dietary wasn’t as restrictive as it has been in the past several years. So I just decided I’m going to get it done.”*
Social influence Family	*“I think I would be more motivated if I had some kind of family history. I don’t have any family history of colorectal cancer.”*		*“Well, that [family and friends] always has influence, but you know, I’m not married, I’m a single man. So it’s not as if I had a wife here to badger me. I don’t.”*	*“The reason it helps me is that my sister had breast cancer. So I just focus on that. They caught hers early enough that she’s a survivor, so I tend not to take tests and stuff for granted.”* *“It’s just what really pushed me over to finally complying, at least this year, was internal pressure, my own pressure on myself and family pressure.”*
Social influence: Doctor	*“If the doctor recommended [screening], based on his or her experience. Or if they said maybe based on research that a certain type [of test] is more effective.”*	*“But if I ran into health issues, let’s say I had blood in my stool or something or I had real irritable bowel, I would definitely talk to my doctor’s office.”*	*“I think the doctor badgering me is about the best thing. I think Dr. O’s a really good doctor and I think he’s really got my interest at heart. I think you can’t hardly beat that.”*	*“…that’s also showing that the doctor cares for you, it’s not only because your arm hurts or something, why you’re going to the doctor, it is beyond that, and looking out for you… It’s preventive.”*
Prevention	*“Well, I know if it’s detected early enough, it’s – what’s the word I want to use? Curable.”*	*“I think again anything that’s an early indicator to let you know if something’s going wrong, and it’s not that hard to do.”* *“Especially with a little bit of a family history, but I think I come down slightly on the side of it’s better to know and deal with it just from having people who died of cancer and various things. Yeah, I think I would want to know. That for me personally isn’t a real barrier, it’s more just details of the test itself.”*	*“I: So it helps that the doctor’s reminding you or mails [the test kit] to you? That’s a good thing that Group Health is doing?* *“P: I think it is. It’s part of what we pay for. Group Health is into some degree of preventive medicine and I think there’s nothing wrong with preventive medicine, we need more of it.”*	*“Oh, I do what I need to do to keep healthy, so yeah, if I can stay ahead of anything that shows up, I always feel we’re ahead of the game. And I need to be able to work until I retire, so I have to stay healthy.”* *“But it helped me say this is preventive, not preventive of colon cancer but early recognition of what could happen on that… just an urging to get something done in time rather than wishing later on that… you had done it earlier…”* *“It’s very dangerous, it’s deadly, and I wouldn’t want to be one of the statistics.”* *“Things that could go wrong…these kinds of things can happen and creep up on you without you knowing.”*

^a^Within the context of receiving a pamphlet about colorectal cancer screening choices and at least two mailed fecal tests

Another important facilitator was *prevention*, or taking care of one’s health through prevention and early detection of disease, which some participants considered especially relevant as one aged. *“Oh, I do what I need to do to keep healthy, so yeah, if I can stay ahead of anything that shows up, I always feel we’re ahead of the game. And I need to be able to work until I retire, so I have to stay healthy.”* Social influence, such as a provider recommendation or family or friends reminding or even pressuring participants to be screened, was also frequently mentioned. One converter stated *““I think the doctor badgering me is about the best thing.”* One never screener stated *“You know, people do not talk about this”* when asked about the influence of others, suggesting a family member, friend, or physician discussion might have been influential.

Even though avoidance and procrastination were discussed by most of the participants, those that had screened by the time of the interviews, particularly those who had screened at least twice, had more solutions to overcoming forgetting or putting off screening. *“I can’t remember what helped [me] to remember, but I don’t leave it on the table. It’s something I put up in the cupboard and then I’ll probably open the cupboard and go – oh, I got to do that!”* (repeater).

### Factors that influence repeat fecal testing

Because our trial is studying long-term adherence to CRC screening, we were particularly interested in hearing about potential barriers and facilitators that could influence repeat fecal testing (Table 4), because participants choosing this test need to do it annually. Barriers to repeat fecal testing were similar to completion of screening in general. A person in the stopped group noted that if they didn’t have problems they might get lax. One converter stated that a barrier would be finding out the test was not as accurate as other tests. Repeaters noted that making the tests harder to do (such as a return to the meat avoiding diet required for stool guaiac tests) would get in the way of repeating fecal testing. Feeling that one was not at personal risk was noted as a barrier (never screener), while learning they were at risk was noted as a potential facilitator (stopped).

**Table 4:** Repeating Fecal Immunochemical (FIT) Testing^a^

	*Never* screeners (*N* = 13)	*Stopped* - screened once before year 3 (*N* = 5)	*Converted* - screened once after year 3 (*N* = 5)	*Repeated* - screened once before year 3 and at least once after (*N* = 18)
Repeated FIT testing: Barriers	*“I mean if you’re just doing a stool sample, to me that’s pretty easy. That is not a big deal…but if I felt like I was more at risk.”*	*“…if I read some study… that said if you’ve taken this thorough test and you’ve been examined you’re good for five years, I would think I don’t need to do this every year because it seems like I’m okay.”* *“I think I would do the colorectal reminder thing once a year whether I was having problems or not, but if I didn’t have any problems, I might be a little lax in making the appointment as soon as I would otherwise…”*	*“The only thing that would encourage me not to do it is if I found out they’re not effective, that they’re a waste of time – then of course I wouldn’t waste my time.”*	*“I think if they made it more difficult to do Like I said that was harder for me to remember to do the other ones because you had to not eat red meat for two days or something like that. This last test was just a piece of cake and was real easy to do. No problem.”*
Repeated FIT testing: Facilitators	*“…because I can do it all at one time and mail it in. I don’t have to stretch it out and watch everything. So this makes it much…easier.* *“…if my doctor told me I need to keep doing it every year, I want to be a little more attentive to what my doctor says,… I will do it.”* *“I think after the first one, it would be a lot easier.”*	*“Having a good result, having a good test would make me want to repeat.”* *“If they told me they thought I was in a high risk category or my personal tests turned up to make it look like I was in a high risk, then I’d want to do it.”*	*“If the doctor’s office or the notifying firm or whoever reminds me, I’ll keep doing it.”* *“I will do it - once I’ve done it and I think it’s a wise thing to do, I will continue to do it. I don’t need any more encouragement! ...Dr. O was enough.”* *“If they can give good results, at least I know what’s going on without having to take a whole lot of time off to do a more intense test, if the results were satisfactory and I didn’t have to go in and do the colon thing.”*	*“If you do it, it’s over and done with and you get your result back and have peace of mind for another year.”* *“Overall, I get peace of mind from it. You do it, they report back that there’s nothing wrong. So I don’t worry about it until the next test. And I appreciate that. I’m also motivated to do it again.”* *“I: Thinking again about the stool tests or any kind of screening test, what kinds of things might help you continue to do them regularly over time?* *“P: Number one would be my age…Also I’m African American, and it runs very high in our people, and other people say the reason why they’re continuing to go ahead and do it, like the test kits or the screening, whatever.”*

^a^Within the context of receiving a pamphlet about colorectal cancer screening choices and at least two mailed fecal tests

Most participants provided responses as to repeat testing facilitators, with the themes being similar to completion of screening in general: convenience and ease of FIT, pressure from their family, doctor encouragement, prevention and taking care of oneself as one got older, knowing one was at increased risk, getting good results, and getting into a routine of doing it every year. However, repeaters had more specific recommendations on ways to get repeat testing done, including keeping the kit by the bathroom, improving the kit (making the paper that catches the stool stronger), and having a competition with a spouse to get it done first. A never screener said that getting through the test once would help them complete a second test.

### Preferences for screening reminder types

We specifically asked participants what types of reminders to complete screening they would like to receive. Answers varied with no particular patterns by group and included just getting the kit in the mail, additional reminders by phone, mail, or e-mail. However, a few reported that getting a reminder through the patient web portal linked to the EHR was a barrier because they had to remember their password to get on the site. Participants thought getting multiple reminders would be acceptable, as it might help to get screening done. Generally up to three reminders was the number participants found acceptable.

When asked about whether it would be OK for a nurse other than one from their clinic to call (a centralized calling system was used in SOS, with two or three medical assistants or nurses calling patients from multiple clinics), they said this was very acceptable as long as the person calling made a personal connection with the patient by being familiar with their records and able to communicate with their providers. They noted that they wanted the centralized program to be coordinated with kits offered in-person at clinic visits. Participants stated that the person calling them should be knowledgeable about CRC screening, clarify the simplicity of the FIT tests, and address any confusion about when colonoscopy might or might not be needed for follow up.

### Barriers and facilitators for CRC screening by subgroup characteristics

We did not find any differences between African Americans, a subgroup of interest because of their lesser responsiveness to SOS stepped interventions, compared to others as to barriers and facilitators to CRC screening initiation, repeating fecal testing, or preferences for reminders, a group of interest because they were. While current smokers accounted for less than one-fourth of the total number of participants (*n* = 10/41), they comprised two-fifths (*n* = 6/18) of the participants who had never screened for colon cancer. Among this group of never-screeners, the barriers to FIT testing mentioned most often by smokers were comorbidities and lack of attention. Of the five never-screeners who talked about other health conditions that prevented them from screening, three were smokers.

### Perspective on potential CRC screening blood tests

Almost all participants in this sample of never and inconsistent screeners had a positive response to the idea of having a blood test for CRC screening, with almost two thirds noting they would prefer it to other forms of testing. Among the 13 never screeners, about three-fourths expressed enthusiasm about blood testing, saying that they preferred it and would likely do it, none said they would not do it. Never screeners tended to use much more enthusiastic language in reacting to questions about blood testing than about other forms of screening with comments such as: *“Oh, I would do that right away”; “In a heartbeat”; “Whoa-it sure would be easier, I would think”; “One hundred percent.”*

The facilitators for blood testing most frequently cited were convenience because they go into the clinic for lab tests for other reasons already: *“Just show up at the lab and take a number, and it’s all done.”* The quickness was a clear advantage. Some noted it would allow them to avoid handling their stool: *“Yes, I would the blood test rather than playing with doodie. I can do that with my grandbabies.”* Another participant noted getting a blood test was more familiar to him than the mailed kit. One participant already thought the stool test *“could not be any easier”* and liked that it could be done in the privacy of one’s home at a time of one’s choosing while blood testing requires going to a clinic.

Participants had different opinions about the potential accuracy of a CRC screening blood test, with some mentioning they wanted it to be at least as accurate as the stool test or to meet a certain threshold of accuracy: *“I mean if it was 40% accurate that would be a different thing. If it were up around 80 or 90, I’d feel pretty comfortable about it.”* Some said they would do whichever test was most accurate: *“If it was the same accuracy I would do the blood test. If the stool was more accurate I would do the stool… [otherwise] why bother having the test?”* One person felt that comparative accuracy was not an issue, “*considering that the colonoscopy itself has better accuracy.”* A few participants (all repeaters) expressed the view that the stool test was more appropriate because it was a test done directly on the area potentially affected.

## Discussion

While our interviews revealed many facilitators and barriers previously reported in other studies, our study is unique in that it explored these topics within the context of an ongoing screening program. All participants were offered at least two mailed fecal kits (all had opportunities to complete guaiac tests and most FIT) or alternatively colonoscopy or flexible sigmoidoscopy. Avoidance and procrastination were cited by all groups as the most common barriers for not screening.

Previous studies have also mentioned aversion to stool as a barrier, including after mailing participants fecal screening tests [14, 20, 21]. Participants in our study were encouraged to choose colonoscopy and flexible sigmoidoscopy if they preferred this to stool testing. However, the barriers for completing colonoscopy, combined with some patients’ perception of colonoscopy as the preferred test, paradoxically kept them from doing any testing. While repeat screeners experienced barriers and facilitators similar to never, stopped, and converted screeners, they provided most of the information on facilitators for screening and overcoming barriers.

Our study also provides insight on factors related to repeat screening, which were similar to screening in general, with convenience and positive test attributes (one sample FIT) seeming to be most important. Participants were in general positive about the SOS mailed program, and no one had concerns about getting reminders from a medical assistant or nurse that served all clinics, but they wanted the nurse to be knowledgeable about CRC screening and their personal circumstances. The group as whole did not object to reminders and in many cases multiple reminders, but were very heterogeneous in the preference for type of reminders.

We also explored the role of CRC blood test screening, which is of particular relevance, because the FDA recently approved a blood test specifically for the purpose of increasing adherence to CRC screening among individuals who are otherwise unlikely to be tested. Never screeners in our sample stated they would be willing to do a blood test. Less clear is whether they also would be willing to do follow-up diagnostic colonoscopy if the blood test was positive.

A recently published systematic review and synthesis of qualitative studies by Honein-AbouHaidar et al., found 94 qualitative studies describing facilitators and barriers to participation in CRC screening [22]. Based on findings from these studies they created a conceptual framework of factors influencing screening uptake, with awareness of CRC screening as a central key component, influencing both CRC screening barriers and facilitators. In this qualitative study participants awareness was not a key theme, except in a few instances where participants, particularly never screeners, mentioned wanting more specific information about CRC and CRC testing. Participants had been made aware of the importance of CRC screening multiple times, including receipt of an introductory pamphlet, a phone call for verbal consent, and at least two rounds of mailings that included facts and narratives about CRC and how screening prevented CRC and CRC deaths. Otherwise, barriers listed in the review, such as fear of a cancer diagnosis or avoidance, were similar to those we found. Fatalism was also described as a key barrier, which we think is a type of avoidance behavior; it allows people to turn their attention away from screening, because of the perception that screening will have little impact on outcomes. Categories of facilitators listed in Honein-AbouHaidar’s review were similar to our study including physician recommendation and self-motivation which was congruent with prevention and taking care of oneself [22]. The review emphasized public education and social networks as other important categories of facilitators. However, in our study these types of facilitators were mentioned less often, probably because participants were part of an ongoing informational and mailed CRC screening program.

Few studies have examined psychosocial constructs related to repeat CRC screening. Palmer et al. conducted focus groups with a diverse group of 128 individuals invited to participate in at least two rounds of the English National Health System (NHS) mailed fecal testing Bowel Cancer Screening Program [23]. Most reported no uptake on at least one occasion. Barriers to participation included aversion to handling stool, completing a diagnostic test at home as opposed to a clinic, wanting to avoid positive results, and feeling well with low perceived relevance. Lack of provider recommendation was another important barrier. Jones et al. noted that patients ranked lack of physician recommendation as the most important barrier to screening among never screeners, overdue, and up-to-date screeners [24]. However, without a mailed fecal testing program, provider order or provision at clinic visits is the only way to get screened. Duncan et al. found that prior history of screening was a strong predictor of subsequent screening, with persistent refusers less likely to have screened prior to the mailed program [15]. Individuals with lower CRC screening self-efficacy (less confidence in their ability to complete screening) were also more likely to refuse and less likely to repeat screening.

In a prior study we reported predictors of uptake and repeating CRC screening on a subset of participants in the SOS study who completed a survey at baseline and at 2 years [13]. The survey included questions on pros and cons of different screening tests, perceived risk, social influence, and self-efficacy to complete screening tests. We found that social influence, perceived risk, pros and cons of specific tests did not predict initiation of or repeat screening; however, both surveys took place before the switch from the 3 sample guaiac test to the 1 sample FIT. Given the number of comments about FIT convenience, the availability of this test likely led to more people initiating or repeating tests by the time of the interviews. Our prior study’s questionnaire did not include questions specific to avoidance, procrastination, or optimism. Vernon (co-author) and others have recognized that prior models for predicting screening behavior needed to be expanded to include this domain, and new tools are now available and tested [25].

Our current study had several limitations. Our sample was small and included patients who had previously provided verbal consent to participate in the ongoing study and interviews, so they may be more engaged than the general population. This group may be different from never and infrequent screeners in the general public. Additionally all the participants had health insurance, with no out of pocket expenses for CRC screening tests (but may not have full coverage for follow-up diagnostic tests). Everyone in our sample also spoke English. We also did not interview participants who screened regularly, who might have provided additional insight on factors that facilitated screening particularly repeat testing. Our study also has strengths: the interviews were comprehensive and were performed with participants who received multiple mailed fecal kits and the option to do endoscopic screening.

SOS is an ongoing study, looking at the impact of the mailed program and stepped intensity intervention on long-term adherence to CRC screening. We previously found that the mailed components doubled adherence to screening over the first 2 years of the trial, while this plus phone assistance, or mailed, phone assistance, plus nurse navigation led to smaller incremental increase. In years 3 and 5 we omitted the phone and navigation components. Many patients eventually got colonoscopy, mostly for screening, but also as follow up of positive fecal tests or symptoms. The remaining group still needed to do kits annually, and a larger proportion had done no testing. In year 6 we brought back the nurse navigator. We used information from this study to refine interventions, in particular providing information to never screeners on the importance of screening and ease of the one-sample FIT test, being mindful of the competing nature of choice and how ambivalence about colonoscopy might impede some individuals from doing any screening. For late screeners, we addressed procrastination and other avoidance behaviors and personalized reminders. Results of these efforts will eventually be available and will inform ways to successfully support people to screen for the first time and, for those choosing fecal testing, screen annually long-term over many years.

## Conclusion

Through analysis of interviews with patients with suboptimal screening adherence, those who chose to never complete CRC screening despite receiving multiple rounds of mailed fecal tests were more likely than others interviewees to report screening barriers including avoidance, dislike of handling stool, competing health concerns, and fear of results or cancers. Participants whom had completed screening at least once were more likely to report screening facilitators including the simplicity of the 1-sample test, convenience, taking care of one’s health, and the influence of health providers, family, and friends. Future CRC screening programs should be designed to minimize these barriers and maximize facilitators to improve long-term screening adherence.

## Introduction

Thanks for agreeing to schedule this interview with me. Is this still a good time to talk? You may recall participating in the Smart Options for Screening (SOS) study in the past. We are trying to understand the views and experiences of patients who may have been previously contacted about colon cancer screening. We will use these interviews to guide future improvements to the SOS colon cancer screening program. Your name and other personal information will be kept confidential, and you can choose not to answer any question that you do not want to

1. Could you tell me about your experience, the last time you considered whether or not to do a screening test for colon cancer?

- *Probe*: How this is different from other cancer screening tests such as breast cancer or prostate cancer?

2) Are you familiar with any screening tests that might help detect colon cancer early? What have you heard about?
  - *Probes:* Are you familiar with a stool test (kit, FIT kit) that screens (or test) for colon cancer? What does this mean to you? How did you hear about it?
  - *Probes*: What have you heard about colonoscopy? What have you heard about flexible sigmoidoscopy?
3) Could you tell me about the last time you can remember getting a stool kit test in the mail?
  - *Probes:* What did you do first? What did you think about it? How did receiving the stool kit make you feel? What did you do with the stool kit in the end? What made you (do it, delay, not do it)?
4) How often do you think stool tests are supposed to be done? If they don’t say yearly, say “Group Health recommends that you do this yearly. How would you feel about doing a stool test every year (once a year)?”
  - *Probes:* How you might want to be reminded, the amount and type of reminders, and getting stool kits in the mail. How many mailings would be too many? What do you think about getting a phone reminder to do a stool kit?
5) What would be a situation where you would NOT complete a stool test (or other test) to screen for colon cancer? Can you tell me more about that?
6) Could you tell me the types of things that would help or motivate someone like you to have a colon cancer screening? Can you say more about that?
  - *Probes*: social influence of family, friend, doctor; risk of colon cancer, fear of colon cancer.
7) Could you tell me the types of things that would keep someone like you from having a colon cancer screening? What gets in the way or slows you down? Can you say more about that? Was cost or insurance coverage an issue?
  - *Probes*: Cost, coverage, time off work, needing help with appointment, getting or doing the prep, help with transportation; different intervals; embarrassment; disgust, fear of colon cancer.
  - *Probe:* Would you find it useful for a nurse at Group Health to help you figure out which costs are covered by your health plan?
8) Who would you trust to get information on or help set up a test for colon cancer screening (like a colonoscopy)? Who else would you trust?
  - *Probe*: How much does it matter to you whether or not the screening staff who calls you is in contact with your personal doctor? In what ways is that important to you? How might that affect whether or not you get screened?
  - *Probe*: Would it be OK to talk to someone who works with your doctor (on your doctor’s team or who could communicate with your doctor)? What types of contact would you want the person you’re talking with to have with your doctor? (If needed, offer examples: send a secured message to your doctor, call your doctor on the phone, set up a phone appointment between you and your doctor, etc.)
9) What kind of things would you want to hear about or get help with, if you got a phone call about colon cancer screening? What kind of things would you NOT want to hear about?
  - *Probe*: Would you prefer to hear more or less about the benefits of getting a screening test? Why? What kinds of things would you like screening staff to say/not say?
  - *Probe:* Would you prefer to hear more or less about your risk of getting colon cancer? Why? What kinds of things would you like screening staff to say/not say?
10) What would be the right number of times to get a call about colon cancer screening for someone like you (in the context of the things they listed, e.g. reminding to do a stool kit, or help with making or keeping a colonoscopy appointment)? How many calls would be too many for someone like you?
11) What would make someone like you decide not to keep doing screening tests after you had done one (or done a few)?
12) What would help someone like you to do repeated colon cancer screening tests over time?
13) What do other people you know, who are around your age, do about getting screened for colon cancer? *Probe*: Do most people your age get repeated screening tests, just one test, or none?
14) If a blood test was to become available as a screening test for colon cancer, how would this influence your decision about being screened?
15) Is there anything else you would like to share?

Thank you so much for talking with me today. I will be mailing you $30.00 today as a thank you.
